# *Parathyroid Hormone* Gene and Genes Involved in the Maintenance of Vitamin D Levels Association with Mandibular Retrognathism

**DOI:** 10.3390/jpm11050369

**Published:** 2021-05-02

**Authors:** Erika Calvano Küchler, Caio Luiz Bitencourt Reis, Guido Marañón-Vásquez, Paulo Nelson-Filho, Mírian Aiko Nakane Matsumoto, Maria Bernadete Sasso Stuani, Maria Angélica Hueb de Menezes Oliveira, Peter Proff, Christian Kirschneck

**Affiliations:** 1 Department of Orthodontics, University of Regensburg, Franz-Josef-Strauss-Allee 11, 93053 Regensburg, Germany; erikacalvano@gmail.com (E.C.K.); Peter.Proff@klinik.uni-regensburg.de (P.P.); 2 Department of Pediatric Dentistry, School of Dentistry of Ribeirão Preto, University of São Paulo, Av. do Café S/N, Ribeirão Preto 14040-904, Brazil; caioluizreis@usp.br (C.L.B.R.); nelson@forp.usp.br (P.N.-F.); manakane@forp.usp.br (M.A.N.M.); bernadete@forp.usp.br (M.B.S.S.); 3 Department of Pediatric Dentistry and Orthodontics, School of Dentistry, Federal University of Rio de Janeiro, R. Prof. Rodolpho Paulo Rocco 325, Rio de Janeiro 21941-617, Brazil; guido_amv@hotmail.com; 4 School of Dentistry, University de Uberaba, R. Frei Paulino 30, Uberaba 38025-180, Brazil; angelicahueb@hotmail.com

**Keywords:** mandible, retrognathism, gene, polymorphism

## Abstract

In this study we evaluated whether single nucleotide polymorphisms (SNPs) in the genes encoding PTH, VDR, CYP24A1, and CYP27B1 were associated with mandibular retrognathism (MR). Samples from biologically-unrelated Brazilian patients receiving orthodontic treatment were included in this study. Pre-orthodontic lateral cephalograms were used to determine the phenotype. Patients with a retrognathic mandible were selected as cases and those with an orthognathic mandible were selected as controls. Genomic DNA was used for genotyping analysis of SNPs in *PTH* (rs694, rs6256, and rs307247), *VDR* (rs7975232), *CYP24A1* (rs464653), and *CYP27B1* (rs927650). Chi-squared or Fisher’s tests were used to compare genotype and allele distribution among groups. Haplotype analysis was performed for the SNPs in *PTH*. The established alpha was *p* < 0.05. Multifactor dimensionality reduction (MDR) was used to identify SNP–SNP interactions. A total of 48 (22 males and 26 females) MR and 43 (17 males and 26 females) controls were included. The linear mandibular and the angular measurements were statistically different between MR and controls (*p* < 0.05). In the genotype and allele distribution analysis, the SNPs rs694, rs307247, and rs464653 were associated with MR (*p* < 0.05). MDR analyses predicted the best interaction model for MR was rs694–rs927650, followed by rs307247–rs464653–rs927650. Some haplotypes in the *PTH* gene presented statistical significance. Our results suggest that SNPs in *PTH*, *VDR*, *CYP24A1*, and *CYP27B1* genes are associated with the presence of mandibular retrognathism.

## 1. Introduction

Skeletal malocclusions are a set of human craniofacial morphologic characteristics that result in an improper skeletal relationship of the jaws and specific facial patterns. There is considerable evidence from family and twin studies suggesting that genes play a significant role in the etiology of skeletal malocclusions [1]. Familial occurrence of skeletal Class II malocclusion has been reported in some studies including twin and triplet studies design and in family pedigrees studies design [1]. A clinical and cephalometric study evaluating 114 patients (48 twin pairs and six sets of triplets) performed an intra- and inter-pair comparison to determine concordance/discordance rates for monozygotic and dizygotic twins. The monozygotic twin pairs presented 100% concordance, while almost 90% of the dizygotic twin pairs were discordant for the Class II division 2 malocclusion [2]. A study evaluating skeletal and dental patterns demonstrated that hereditary factors were responsible for 40% of the variations resulting in malocclusion and the genetic component was higher for skeletal than for dental patterns [3]. In a classical study, cephalometric radiographs of several families were analyzed to evaluate facial morphologic differences among family members. The authors observed a high correlation between parents and their offspring and concluded that a strong familial tendency for skeletal malocclusions exists, supporting the impact of genetic background on face morphology [4]. More recently, many studies in different populations have been performed and demonstrated that genes involved in a variety of functions are associated with skeletal malocclusions and morphological patterns of the face [5,6]. Mandibular retrognathism (MR) is a common skeletal malocclusion in humans. It refers to a retruded position of the mandible as a consequence of an anomaly of the skeletal jaw–cranial base relationship. This unfavorable positional relationship of developing jaws is diagnosed through image exams [7].

Single nucleotide polymorphisms (SNPs) are a type of genetic variant involving variation of a single base pair in the genome. This is the most common type of genetic variation in humans, and it has been stated that these variants could explain differences in individual predisposition to present complex traits [8]. Previous studies have shown that MR was associated with SNPs in various genes such as *Myosin 1H* (*MYO1H*) [9], *Matrilin 1* (*MATN1*) [7], *ADAM Metallopeptidase with Thrombospondin Type 1 Motif 9* (*ADAMTS9*) [10], and *Bone Morphogenic Protein 2* (*BMP2*) [11] genes. In addition, SNPs in the gene encoding growth hormone receptor (GHR) have been associated with variations in the mandibular pattern including prognathism [5,12], supporting that genes coding for hormones, hormone receptors, hormone precursors, and molecules involved in hormonal synthesis could also be involved in the etiology of other phenotypes. 

Two important hormones that play a crucial role in bone development are parathyroid hormone (PTH) and vitamin D (a secosteroid hormone). These are major regulators of mineral metabolism involved in calcium and phosphate homeostasis as well as in bone growth and development [13]. The biological actions of vitamin D are exerted by binding to the nuclear vitamin D receptor (VDR) [14]. VDR is expressed in the parathyroid glands acting as sensors for the detection and maintenance of adequate vitamin D levels, regulating PTH synthesis and release [15], which among other tissues affects the periodontal ligament [16]. Additionally, other molecules such as the vitamin D 24-hydroxylase (CYP24A1) and 1-hydroxylase (CYP27B1) enzymes participate in related processes, being considered as pivotal determinants of the local concentration of active vitamin D [17]. 

Nutrition and biomechanical factors can affect the facial pattern, however, the most important factor related with craniofacial growth and development seem to be hormones, genetic, and molecular mechanisms as well as the interplay among them [18]. There is some evidence from animal model studies that PTH is involved in mandible and mandibular condyle development and mineralization [19,20]. Additionally, a study in rats using cephalometric analysis to investigate the effects of a low calcium and vitamin D-deficient diet on craniofacial morphology and growth observed that vitamin D-free and low calcium diet affects mandible development [21]. However, the role of PTH, vitamin D, and genes associated with these two hormones in the human MR is still unclear. Therefore, in the present study, we evaluated if SNPs in genes encoding *PTH*, *VDR*, *CYP24A1*, and *CYP27B1* as well as the interplay among them were associated with MR. A tightly controlled connection between vitamin D, serum calcium, and genes involved in the maintenance of vitamin D levels orchestrates mineral homeostasis and development.

## 2. Materials and Methods

### 2.1. Sample

This nested case-control study was previously approved by the Human Ethics Committee of the University of São Paulo—Ribeirão Preto Dental School (# 01451418.3.0000.5419). Informed consent/assent was obtained from all participants and/or their legal guardians where age-appropriate. This project was performed according to the Helsinki Declaration and its amendments. The Strengthening the Reporting of Genetic Association study (STREGA) statement checklist [22] was followed to develop and report the results of this study.

Patients undergoing orthodontic treatment at the University of São Paulo were recruited and consecutively included in this study from 2015 to 2017. Patients with syndromes, congenital alterations, hormonal, and/or metabolic disorders or those with previous orthodontic and/or orthopedic treatments were not included. None of the patients reported history of vitamin D deficiency. All patients included were Brazilian biologically-unrelated and self-reported as Caucasian. Additionally, none of the patients were using vitamin D supplementation and all patients were from the Ribeirão Preto area, which presents a high total sunshine hour and UVB incidence over the entire year.

### 2.2. Phenotypes Definition

**Figure 1:**
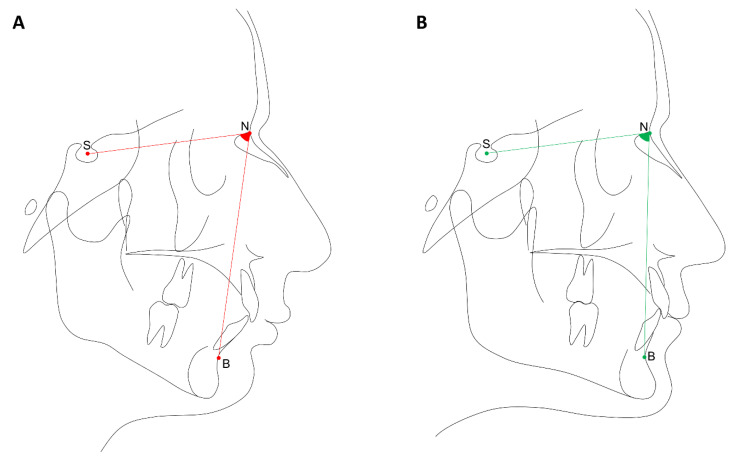
Cephalometric tracings. (**A**) Exemplification of retrognathism (SNB > 78°). (**B**) Exemplification of orthognathic mandible (SNB ranging from 78° to 82°).

Pre-orthodontic lateral cephalograms with the mandible in centric relationship were used and digital cephalometric tracings performed by a calibrated orthodontist using the software Dolphin Imaging Version 8.0 (Dolphin Imaging, Chatsworth, CA, USA), as demonstrated in Figure 1. The following landmarks were used to determine the phenotype: point A, point B, sella (S), and nasion (N) and, therefore, the angular measurements SNB and ANB were calculated. Patients having a retrognathic mandible (SNB < 78°) were selected as cases, and those with an orthognathic mandible (SNB = 78°–82°) were selected as controls. Patients with mandibular prognathism (SNB > 82°) were excluded.

Additionally, the linear measurements associated with mandibular size (mandibular length, Co-Gn; length of mandibular base, Go-Pg; and mandibular ramus height, Co-Go) were measured in millimeters (mm) and compared between the patients with retrognathic mandible and the patients with orthognathic mandible.

### 2.3. Allelic Discrimination

Genomic DNA extracted from saliva was used for genotyping analysis. Briefly, for saliva collection, saline mouth solution to rinse in the mouth for 60 s was used. Therefore, the genomic DNA was extracted from buccal epithelial cells from saliva samples as previously described [23]. Quantification of the concentration and purity of the DNA was determined by spectrophotometry (Nanodrop 1000; Thermo Scientific, Wilmington, DE, USA). 

Six SNPs were evaluated in the present study and are reported in Table 1. The genotyping was blindly performed using the Taqman™ method for real-time PCR in the StepOnePlusTM sequence detection system, Applied Biosystems™ (Foster City, CA, USA) or in the Mastercycler® ep realplex-S thermocycler, Eppendorf AG (Hamburg, Germany). Additionally, 10% of the sample was genotyped twice and an agreement of 100% was observed. The reaction was previously described in [24].

### 2.4. Statistical Analysis

The Chi-squared test was used to estimate the Hardy–Weinberg equilibrium (https://wpcalc.com/en/equilibrium-hardy-weinberg/) (accessed on 2 April 2021).

Chi-squared or Fisher’s exact tests were used to compare gender, genotypic, and allelic distribution among groups. The Mann–Whitney U test was applied for the comparison of continuous data among the groups after evaluating the normality by the Shapiro–Wilk test, and Levene’s test was used to assess the homogeneity of variance. These analyses were performed by GraphPad Prism version 7.0 for Windows (GraphPad Software, San Diego, CA, USA).

Haplotype analysis was performed for the SNPs in PTH by PLINK version 1.06 (https://zzz.bwh.harvard.edu/plink/ld.shtml) (accessed on 2 April 2021). The established alpha for these analyses was *p* < 0.05.

Multifactor dimensionality reduction (MDR) [31] was done to identify SNP-SNP interactions using gender and age as co-variables as previously reported [11]. Two specific software programs (Multifactor Dimensionality Reduction 3.0.2 [31], and MDR Permutation Testing Module 1.0 beta 2 [32], available in sourceforge.net/projects/mdr/files (accessed on 2 April 2021)), were used to perform a 10-fold cross-validation consistency (CVC), testing balancing accuracy (TBA), and the 1000 permutation test to determine the statistical significance of the models. Models with the cross-validation consistency of 9/10 or 10/10 and the TBA > 0.55 and *p* ≤ 0.05 were considered as the best models. Entropy values were calculated according to Jakulin and Bratko [33], and MDR created dendrograms and interaction graphs using these values. MDR analysis can be performed freely in a JAVA language.

## 3. Results

**Figure 2:**
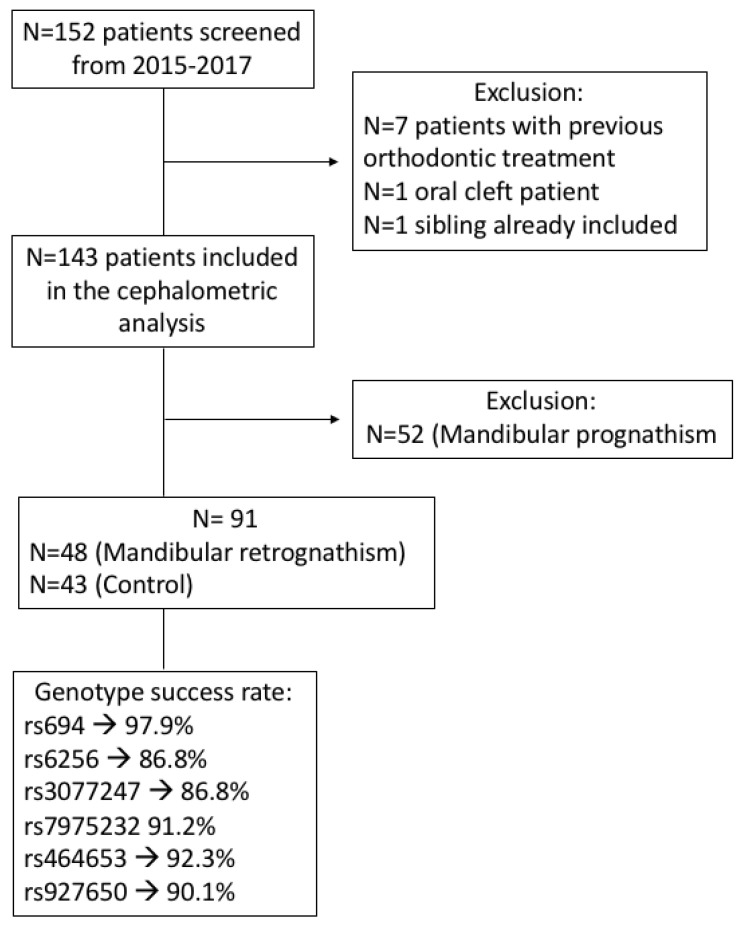
Flow chart of patient selection.

A total of 91 patients were included. Figure 2 presents the flow diagram of the study participants and genotype success rate for each SNP. The sample characteristics and mandibular parameters according to the groups (MR and control) are shown in Table 2. There was no significant difference between the groups according to age and gender distribution (*p* > 0.05). The participants in the MR group had significantly lower linear measurements in the mandible than the control group (*p* < 0.05). Furthermore, the SNB angle was significantly lower and the ANB angle was significantly higher in the retrognathic subjects (*p* < 0.001).

All SNPs assessed were within the Hardy–Weinberg equilibrium. Genotype and allele distributions are demonstrated in Table 3. The SNPs rs694 and rs307247 in *PTH* and the SNP rs464653 in *CYP27B1* were significantly associated with mandibular retrognathism (*p* < 0.05).

The best models for MDR analyses were rs694 (*PTH*), rs927650 (*CYP24A1*), rs307247 (*PTH*), rs464653 (*CYP27B1*), rs927650 (*CYP24A1*) and rs694 (*PTH*), rs6256 (*PTH*), rs307247 (*PTH*), rs7975232 (*VDR*), rs464653 (*CYP27B1*), and rs927650 (*CYP24A1*) with a cross-validation consistency of 10 out of 10. Table 4 shows the MDR-predicted interaction models.

**Figure 3:**
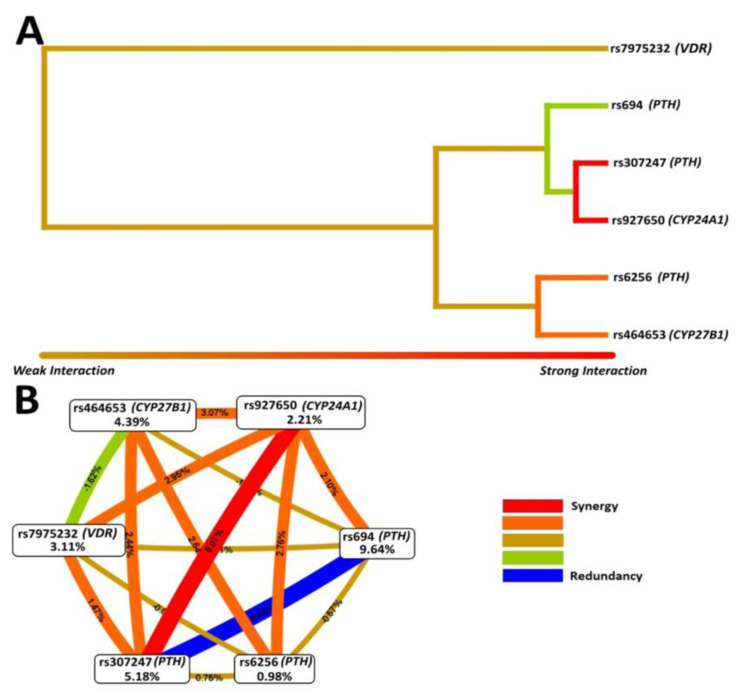
Interaction analysis for the studied SNPs by MDR. (**A**) Interaction dendrogram. Dendrogram shows keys connecting the SNPs. Strongly interacting SNPs are connected by keys farther to the right, whereas the weakly interacting SNPs are connected by keys farther to the left. (**B**) Interaction entropy graph. Each SNP are represented by a node and percentages inside the nodes are their entropy values. The percentages between the nodes indicate the entropy values resulting from the combination between SNPs.

Figure 3 shows the interactions between SNPs (dendrogram and interaction map). The strongest synergism interaction effect was between rs307247 (*PTH*) and rs927650 (*CYP24A1*), followed by the interaction between rs6256 (*PTH*) and rs464653 (*CYP27B1*).

The haplotype analysis for the SNPs in the *PTH* gene is presented in Table 5. Some haplotypes were associated with mandibular retrognathism.

## 4. Discussion

Disorders of the face and dental jaws such as skeletal malocclusions are very common developmental disorders in all ethnic populations [34]. Skeletal malocclusion affects dental and facial tissues [34]. Mandibular retrognathism is a condition that not only affects facial aesthetics, but is also associated with problems such as temporomandibular disorders [35] and alterations in the respiratory pattern and normal sleep [34,36]. The prevalence of skeletal malocclusion ranges in different ethnic groups [34]. In the orthodontic population studied here with evaluated Brazilian patients, skeletal class II malocclusion affects about 30% of the sample [11,37] and mandibular retrognathism affects 35% of the sample [8]. Retrognathism is a much more frequent condition than mandibular prognathism; however, its etiology has been the subject of only a few studies. Most studies on the genetic background of skeletal malocclusion have focused on prognathism and Class III malocclusion, which is less frequent in the general population. Studies on genes involved in skeletal Class II malocclusion, retrognathism, and micrognathia are rarer [2] and only a few genes and SNPs have been evaluated thus far [7,9,10,11,38,39]. Therefore, the present study aimed to explore the role of some SNPs as well as their interaction in the mandibular retrognathism phenotype in humans. 

Sequence variation in human genes is largely confined to SNPs and is valuable in tests of association with common traits such as retrognathism. Uncovering the SNPs and genes responsible for the regulation of facial morphology is not a trivial task. Human facial development is a complex multistep process, implicating several signaling cascades of factors. The mechanisms involved in this process include the expression of innumerous genes and protein translation. These events are precisely timed and are under hormonal control [40]. Therefore, in the present study, we evaluated whether common SNPs in genes involved in hormonal synthesis and metabolisms were associated with mandibular retrognathism.

PTH is an 84-amino acid peptide hormone synthesized in the cells of the parathyroid glands. This hormone is a major mediator of bone remodeling and plays a crucial role in calcium homeostasis, showing several effects on the bone remodeling process, resulting in anabolic activity (bone formation) and catabolic activity (bone resorption) [41,42]. PTH promotes calcium release at the bone level, in which a hypocalcemic signal will lead to a higher release and synthesis of PTH, restoring the serum calcium to normal [43]. In our study, two SNPs in the gene encoding PTH were associated with retrognathism in the univariate analysis: the intronic SNP rs694 and the SNP rs307247, which is located in a 3’ untranslated region (3’UTR). One important aspect in genetic association studies is that the majority of traits were associated with non-coding regions (intronic and intergenic) called regulatory SNPs [44]. Intronic variants can impact alternative splicing by interfering with splice site recognition [45]. Additionally, 3’UTRs can modify gene expression by controlling the mRNA nuclear export, cytoplasmic localization and stability, or by affecting translational efficiency. These gene fragments are targeted by microRNA as well as regulatory molecules [46]. However, one aspect to be highlighted for future studies designs is that a redundancy was observed between rs694 and rs307247 in the interaction map.

In our study, we also evaluated the SNP rs6256 located in exon 3 of the *PTH* gene. This nonsynonymous variant might contribute to the altered gene expression. A previous study demonstrated that serum PTH levels were higher in individuals carrying the rs6256 AA genotype [26]. We observed that this SNP was associated with MR in the MDR analysis and in the haplotype analysis.

It is well known that vitamin D requires the involvement of several key proteins in a closely regulated process. The vitamin D produced in the skin, in response to UVB light or from exogenous supplements, is sequentially hydroxylated into the active metabolite 1,25-Dihydroxyvitamin D (1,25(OH)2D, the biologically active form of vitamin D) in the kidney and other tissues via the enzyme CYP27B1 [47]. Additionally, 1,25(OH)2D concentrations are regulated by CYP24A1 [48]. In the genotype distribution as well as in the MDR analysis, the intronic SNP rs464653 in *CYP27B1* was associated with mandibular retrognathism. A strong interaction and synergism were observed between the SNP rs307247 in *PTH* and rs927650 in *CYP24A1*. Parathyroid glands contain CYP27B1 and CYP24A1 and the expression levels of both these enzymes are transcriptionally regulated by PTH [49]. Therefore, our results suggest that SNPs in genes involved in this highly regulated interaction are involved in mandibular morphology in humans.

A study evaluating animals fed with a low calcium and vitamin D-deficient diet observed that this diet caused alterations in craniofacial morphology including reduced mandibular dimensions [21]. The biological effects of vitamin D are mediated by binding to its receptor, a member of the nuclear receptor superfamily, encoded by the gene *VDR* [14]. Although the studied SNP rs7975232 in *VDR* seems to only have a small role in the mandibular retrognathism phenotype, this result should be interpreted with caution. In our analysis, only one SNP was evaluated in *VDR*, which is a gene with some well-known SNPs. Therefore, the coverage of the gene was a limitation of our study. It is possible that other SNPs in *VDR* as well as their interaction are also involved in the retrognathism phenotype. Additionally, the generalizability of our results to populations of other ancestries is unknown. Further studies are necessary to evaluate the role of these SNPs in skeletal malocclusions in different populations.

## 5. Conclusions

Briefly, MR is a polygenic trait. SNPs in coding and regulatory regions can lead to different gene activities and possible contributions to interindividual variability including variability in facial morphology. Our results support that genes involved in the maintenance of vitamin D levels are involved in the etiology of human MR.

## Figures and Tables

**Table 1:** Characteristics of the studied SNPs.

Gene	SNP	Base Change ^#^	Functional Consequence ^#^	Clinical Significance ^†,‡^	Biological Effects (Reference)
*PTH*	rs694	C > T	Intron Variant	Benign	Low levels of PTH serum [25]
	rs6256 *	G > T	Stop Gained *	Benign	Low levels of PTH serum [26]
	rs307247	G > A	3′ untranslated region	Benign	Low levels of PTH serum [27]
*VDR*	rs7975232 ^#^	C > A	Intron Variant	Benign	High levels of Vitamin-D serum [28]
*CYP27B1*	rs464653	A > G	Intron Variant	Benign	Oral Neoplasm [29]
*CYP24A1*	rs927650	C > T	Intron Variant	Uncertain Significance	High levels of Vitamin-D serum [30]

Note: * (Arg) > (Ter). ^#^ known as Apal. Information was obtained in ncbi.nlm.nih.gov/snp/ (^#^) (accessed on 22 December 2020), ncbi.nlm.nih.gov/CBBresearch/Lu/Demo/LitVar/# (^†^) (accessed on 22 December 2020) and varsome.com (^‡^) (accessed on 22 December 2020).

**Table 2:** Comparison of cephalometric variables between mandibular retrognathism and control groups.

Variables	MR	Control	*p*-Value
Gender n (%)			
Male	22 (45.8)	17 (39.5)	0.672
Female	26 (54.2)	26 (60.1)	
Age			
Median (IQR)	12.0 (4.0)	12.0 (4.5)	0.809
SNB (°)			
Median (IQR)	76.1 (3.5)	80.0 (2.0)	<0.001 *
ANB (°)			
Mean (SD)	4.2 (2.3)	2.4 (2.3)	<0.001 *
Co-Gn (mm)			
Mean (SD)	110 (8.0)	115 (9.4)	0.008 *
Go-Pg (mm)			
Median (IQR)	64.4 (6.9)	68.2 (5.5)	0.014 *
Co-Go (mm)			
Median (IQR)	54.0 (8.8)	56.8 (7.4)	0.044 *

Note: IQR means interquartile range; SD means standard deviation. MR means mandibular retrognathism. * means statistically significant difference (*p* < 0.05).

**Table 3:** Genotype and allele distribution among the groups.

*Gene*	*SNP*		*Frequency-n (%)*			*p-Value*	*OR (CI 95%)*
		*Genotype/Allele*		*Control*	*MR*		
*PTH*	rs694	Genotype	TT	4 (10.8)	19 (44.2)	Reference	-
			CT	22 (59.5)	19 (44.2)	0.004 *	0.18 (0.06–0.59)
			CC	11 (29.7)	5 (11.6)	0.001 *	0.09 (0.02–0.44)
		Allele	T	30 (40.5)	57 (59.4)	Reference	-
			C	44 (59.5)	39 (40.6)	0.014 *	0.46 (0.25–0.87)
	rs6256	Genotype	GG	30 (81.1)	32 (76.2)	Reference	
			GT	7 (18.9)	9 (21.4)	0.740	1.20 (0.39–3.39)
			TT	0 (0.0)	1 (2.4)	>0.999	-
		Allele	G	67 (90.5)	73 (86.9)	Reference	-
			T	7 (9.5)	11 (13.1)	0.472	1.44 (0.51–3.71)
	rs307247	Genotype	GG	12 (33.3)	26 (60.5)	Reference	
			AG	14 (38.9)	12 (27.9)	0.074	0.39 (0.14–1.16)
			AA	10 (27.8)	5 (11.6)	0.019 *	0.23 (0.06–0.78)
		Allele	G	38 (52.8)	64 (75.0)	Reference	
			A	34 (47.2)	22 (25.0)	0.003 *	0.37 (0.19–0.73)
*VDR*	rs7975232	Genotype	AA	14 (35.0)	18 (41.9)	Reference	
			AC	17 (42.5)	21 (48.8)	0.934	0.96 (0.37–2.44)
			CC	9 (22.5)	4 (9.3)	0.121	0.34 (0.10–1.31)
		Allele	A	45 (56.2)	57 (66.3)	Reference	-
			C	35 (43.8)	29 (33.7)	0.184	0.65 (0.34–1.20)
*CYP27B1*	rs464653	Genotype	AA	13 (33.3)	25 (55.6)	Reference	-
			AG	21 (53.9)	15 (33.3)	0.037 *	0.37 (0.13–0.93)
			GG	5 (12.8)	5 (11.1)	0.358	0.52 (0.12–2.09)
		Allele	A	47 (60.3)	45 (64.3)	Reference	-
			G	31 (39.7)	25 (35.7)	0.613	0.84 (0.44–1.68)
*CYP24A1*	rs927650	Genotype	CC	17 (44.7)	15 (34.1)	Reference	-
			CT	16 (42.1)	26 (59.1)	0.197	1.84 (0.75–4.69)
			TT	5 (13.2)	3 (6.8)	0.633	0.68 (0.16–3.45)
		Allele	C	50 (65.8)	56 (63.6)	Reference	-
			T	26 (34.2)	32 (36.4)	0.773	1.09 (0.57–2.12)

Note: * means statistically significant difference (*p* < 0.05).

**Table 4:** Summary of MDR analysis results.

Locus Number	Best Combination	CVC ^#^	TBA ^†^	*p*-Value ^‡^
2	rs694 (*PTH*), rs927650 (*CYP24A1*)	10/10	0.6742	0.040 *
3	rs307247 (*PTH*), rs464653 (*CYP27B1*), rs927650 (*CYP24A1*)	10/10	0.7651	<0.001 *
4	rs307247 (*PTH*), rs7975232 (*VDR*), rs464653 (*CYP27B1*), rs927650 (*CYP24A1*)	8/10	0.7016	0.009 *
5	rs694 (*PTH*), rs307247 (*PTH*), rs7975232 (*VDR*), rs464653 (*CYP27B1*), rs927650 (*CYP24A1*)	8/10	0.6832	0.026 *
6	rs694 (*PTH*), rs6256 (*PTH*), rs307247 (*PTH*), rs7975232 (*VDR*), rs464653 (*CYP27B1*), rs927650 (*CYP24A1*)	10/10	0.7085	0.008 *

Note: * means statistically significant difference (*p* < 0.05). ^#^ The 10-fold CVC indicates a prediction error. ^†^ TBA values show the proportion of individuals correctly classified as the case or control. ^‡^ *p*-values were based on the 1000 permutations test by a specifically developed software for MDR analysis. More details can be consulted in Pattin et al. (2009) [32].

**Table 5:** Haplotype association analysis of SNPs in the PTH gene.

SNPS	Haplotype	MR	Control	*p*-Value
rs694, rs6256, rs307247	C-G-A	0.25	0.47	0.004 *
	C-T-G	0.01	0.03	0.510
	T-T-G	0.11	0.06	0.231
	C-G-G	0.06	0.09	0.530
	T-G-G	0.54	0.34	0.010 *
rs694, rs6256	T-T	0.13	0.09	0.472
	C-G	0.33	0.59	0.001 *
	T-G	0.53	0.31	0.004 *
rs694, rs307247	C-A	0.25	0.47	0.004 *
	C-G	0.08	0.12	0.365
	T-G	0.66	0.40	0.001 *
rs307247, rs6256	A-G	0.26	0.47	0.006 *
	G-T	0.13	0.09	0.510
	G-G	0.60	0.43	0.027 *

Note: * means statistically significant difference.

## Data Availability

The data generated in the present study are included within the manuscript.
